# Occurrence and Fate of Triclosan and Triclocarban in Selected Wastewater Systems across Durban Metropolis, KwaZulu-Natal, South Africa

**DOI:** 10.3390/ijerph19116769

**Published:** 2022-06-01

**Authors:** Babatunde Femi Bakare, Gbadebo Clement Adeyinka

**Affiliations:** Department of Chemical Engineering Mangosuthu, University of Technology, UmLazi 4031, South Africa; bfemi@mut.ac.za

**Keywords:** wastewater, antimicrobial agents, triclosan, triclocarban, contaminants, Durban

## Abstract

Triclosan (TCS) and triclocarban (TCC) are antimicrobial agents that have been used in personal care and consumer products in the past decades. In this study, influent, effluent, and sludge samples collected in selected wastewater treatment plants across the Durban metropolis were qualitatively and quantitatively investigated. It was revealed that the concentration of TCS ranged from 1.906 to 73.462 µg/L, from 1.732 to 6.980 µg/L, and from 0.138 to 2.455 µg/kg in influent, effluent, and sludge samples, respectively. The concentrations of TCC were found to be between 0.320 and 45.261 µg/L, <LOQ–1.103 µg/L, and from 0.107 to 8.827 µg/kg in the influent, effluent, and sludge samples, respectively. Higher concentrations of TCS as compared with TCC were observed in the aqueous samples. However, the concentrations of TCC in the sludge samples were significantly higher than the level of TCS. More water solubility of TCS could be responsible for the observed trend in the influent and effluent samples, while the trend observed in the sludge could be due to the more hydrophobicity character of TCC. The results of this study indicated that substantial amounts of TCS and TCC are been removed during the treatment process which could be a major reason for the decline in the levels recorded in the effluent samples, therefore, reducing the amount of the TCS and TCC that would eventually end up in the surface rivers. Qualitative analyses of the samples indicated the presence of caffeine, tert-butylhydroquinone, chloroxylenol, phenol, 4-(1,1,3,3-tetramethyl butyl), and dimethyl-bisphenol A. Further investigative ecological risk assessment studies are crucial due to the potential threat the contaminants may pose to aquatic lives and humans.

## 1. Introduction

Wastewater systems are the primary source of pollution via which pollutants find their way into surface water systems. The pollution associated with wastewater channels is mostly of anthropogenic origin that could result from domestic, industrial, agrochemical, and pharmaceutical sources or a combination of those, which form a major point source of surface or underground water pollution [1,2]. It is pertinent to note that most of the waste emanating from these anthropogenic sources is raw waste, which in most cases lacks any proper treatment process from the original point source prior to being released into a wastewater treatment channel where it would undergo further treatment processes before being finally released into a surface water system. Due to the heterogeneous nature of wastewater, wastewater systems carry various arrays of pollutants through their influent (domestic, industrial, agrochemical, and pharmaceutical) that are complex to handle by many of the wastewater treatment plants (WWTPs). Given this, it is very difficult for many WWTPs to have zero pollution from their effluent before being discharged into a receiving water body. As a result, many xenobiotic pollutants could find their way back into the surface water, thereby, resulting in an imbalance or reducing the survival rate of aquatic lives. Studies have shown that WWPTs are generally designed to remove oxygen demand (OD), suspended material (SM), pathogenic organisms (PO), and nutrients such as ammonia, phosphate, nitrate, and chloride [3]. There is less focus on the treatment of trace contaminants of emerging concerns (CECs) such as xenobiotic and heavy metals and it is not typically a design criterion for most WWTPs. Generally, most of the conventional treatment processes of municipal wastewater that use short retention times such as trickling filters have been reported to have relatively low removal efficiency (generally <72%) for TCS and TCC [4,5,6], while those with long hydraulic retention times such as activated sludge have been proven to be more effective for TCS and TCC removal [7]. Although attention has been focused on ongoing efforts in future plants, there may be a need to optimize the removal efficiencies of these pollutants [3,8]. 

**Figure 1:**
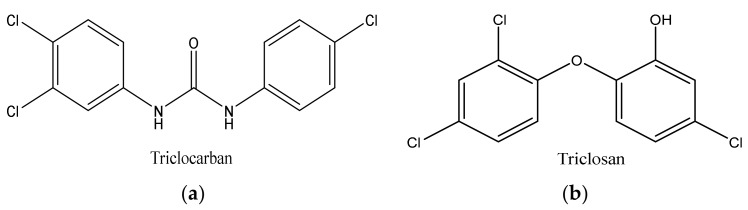
Structure of triclocarban (**a**) and triclosan (**b**).

Triclosan (5-chloro-2-[2.4-dichloro-phenoxy]-phenol (TCS) and triclocarban (N-(4-chlorophenyl)-*N*′-(3,4-dichlorophenyl) urea) (TCC) (Figure 1a,b), are both antimicrobial, bactericide agents. They are phenolic chlorinated compounds with ether functional groups. These antimicrobials are present in most consumer products, in particular, in Europe and Japan, they are commonly added to household soaps, detergents, toothpaste, disinfectants, cosmetics, and medical disinfectants for killing/inhibiting microbes, at levels of up to 0.1–0.3% (*w*/*w*) and 2% (*w*/*w*) of TCS and TCC, respectively [9,10,11,12,13,14]. The maximum allowable concentration of TCS in soap and deodorant in the USA is 0.3%. Biosolids such as organic fertilizer in agricultural lands continue to be used as a secondary source of manure to boost crop yield, therefore, increasing the point source of TCS and TCC in aquatic environments through runoff or leaching [11,12,15]. The tolerable concentration of products containing TCS in South Africa is set at approximately between 0.2 and 0.3% [16,17]. The Food and Drug Administration (FDA) banned the usage of TCS in household products such as liquid soap, gel, foam and bar in the United States in September 2016 and the banning of all human hygiene biocidal products by the European Union was started in January 2017 [18]. Similarly, the FDA has issued a final rule establishing that 19 specific ingredients including TCS and TCC were generally no longer recognized to be safe and effective, therefore, companies have been prohibited from marketing soaps as antibacterial that contain one or more of these ingredients [19]. Research has shown that TCS is a “down the drain” contaminant that is transported in domestic sewage to municipal WWTPs, thereby, ending up in the wastewater effluents, and finally in the aquatic environment, in most cases in surface water, groundwater, or partitioned into the soil or sediment [6]. Triclosan and triclocarban are relatively hydrophobic compounds with octanol/water coefficients (log Kow) of 4.8 and 4.9, respectively [13]. On the one hand, TCS has high water solubility (10.0 mg/L) with a pKa of 7.9, which enables it to be transformed into various other compounds through biotic and abiotic processes and mechanisms within organisms and WWTPs, as well as in the environment [20]. In this context, various aqueous environmental factors such as a change in water pH, temperature, photolytic process, and humidity, as well as living microorganisms (microalgae, fungi, microbacteria, and protists) are capable of degrading parent TCS into a more harmful daughter by-product such as chlorinated phenoxyphenols, chlorinated phenols, and trihalomethanes. On the other hand, due to its molecular nature, TCC remains unionized in a broad range of pH (>8) values (pKa = 12.7) and displays a more limited activity as compared with TCS [20,21,22]. Studies have shown that there are levels of these antimicrobial agents found in wastewater, surface water, and gray water systems, as well as in soil samples. Almqvist and Hanæus [23] reported levels of TCS in gray water systems from Swedish households in the range of 0.075 µg/L–16.6 µg/L. The fate of TCS and TCC in WWTP influents and effluents have been reported by Bedoux et al. [24], and Tran et al. [25] at levels ranging from 1.3 to 86,200 ng/L and from 3.1 to 5370 ng/L for TCS and TCC, respectively. In addition, in South Africa, Lehutso et al. [17] reported levels of TCS and TCC found in the influent, effluent, and sludge samples collected in selected WWTPs across Gauteng Province, South Africa. The levels of TCS found in influent samples ranged between 2.01 and17.6 mg/L and the effluent was between 0.990 and 13.0 mg/L, while the raw sludge was reported to be between 3.65 and 15.0 mg/kg. The concentrations of TCC were 0.0860–2.84 mg/L, <LOD–1.89 mg/L, and 3.65–11.8 mg/kg for influent, effluent, and raw sludge, respectively.

The potential health risks of TCS and TCC to human and animal health have been documented [26,27,28]. Recently, triclosan and triclocarban have gained attention because they have been considered to be an endocrine disruptor, which is capable of influencing reproductive functions [29,30]. These compounds are also considered to be emerging endocrine disruptors, which have the potential to cause health-related issues such as immune dysfunction, affect human reproductive outcomes [10,31,32], and are toxic to aquatic organisms such as algae, fish, and invertebrates [33,34,35].

Apart from personal care products that constitute a threat to aquatic bodies, inorganic pollutants such as the excessive release of nutrients including ammonia, phosphate, chlorides, and nitrous oxide, as well as heavy metals are also inimical to the survival of aquatic biotas. Regrettably, the public is unaware of the associated hazards. Human activities have contributed significantly to the pollution loads of surface waters, which are the main sources of water for larger populations within their catchment areas. The overbearing effects of surface water pollution due to human activities have led to water stress and have contributed significantly to the inadequate supply of water for aesthetic use. This could also have direct impacts on the ecological and corresponding health effects on aquatic lives and humans. Therefore, to protect aquatic ecosystems, as well as drinking water supplies, it is important to examine the fate of TCC, TCS, nutrient concentrations, and other wastewater physicochemical parameters following release to the environment, which was the aim of this study. The main objectives of this study were: to evaluate the levels of nutrients and other physicochemical properties in the influent and effluent samples collected across the Durban metropolis; to quantify the levels of TCS and TCC in the influent, effluent, and sludge samples; to qualitatively determine other contaminants of concern present in the wastewater samples.

The data generated and the results of this study would provide the much-needed information that could be useful for the relevant agencies on the levels and fate of targeted pollutants. Due to the crucial nature of this study’s subject matter, the results should propel further studies to identify the best possible remediation approach for targeted and other potential emerging contaminants in wastewater, and therefore, to safeguard the receiver surface water along with the point of their discharge.

## 2. Materials and Method

### 2.1. Chemicals

Triclosan and Triclocarban standards were purchased from Sigma-Aldrich^®^, South Africa; anhydrous sodium sulfate, organic solvents HPLC grade dichloromethane, and acetone (organic solvent) were supplied by DLD Scientific, South Africa. Glass fiber filters (GF/F, pore size 0.45 μm) were used. Sample interference is a problem in an analytical analysis and, as such, could jeopardize the integrity of the process. The glassware was soaked in an acid bath with tap water for a 24 h duration; the use of detergent or soap was carefully avoided because these could introduce the targeted analytes (TCS and TCC) into the samples, thereby, leading to an overestimation of the TCS and TCC. The glassware was removed from the acid bath, rinsed with running tap water, and then with deionized water; the glassware was finally rinsed with acetone and placed on a rack to dry. The dried glassware was placed in an oven set at 135 ℃ overnight for further sterilization prior to use. This was done to eliminate any possible carryover effect and contaminations during sample preparations.

### 2.2. Description of the Study Location

**Figure 2:**
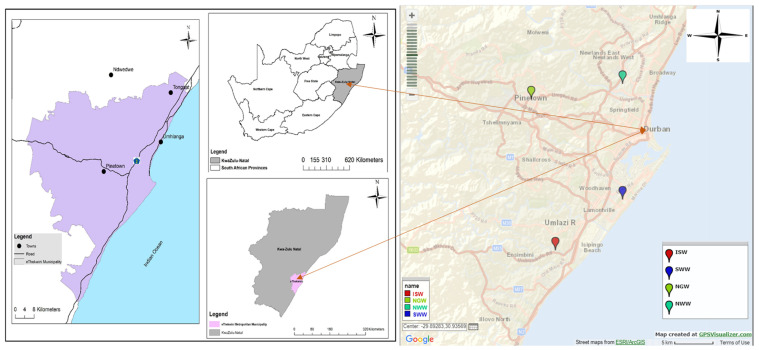
Map showing site locations with wastewater treatment plants across the Durban Metropolis.

The study areas were: the Isipingo Wastewater Treatment Works (29°59′24.936″ S 30°54′21.78″ E), Southern Wastewater Treatment Works (29°55′51.456″ S 30°59′53.268″ E), Northern Wastewater Treatment Works (29°48′49.356″ S 30°52′23.7″ E), and New Germany Wastewater Treatment Works which is known as an innovative water treatment (29°47′43.44″ S 30°59′53.268″ E). The global positioning system (GPS) was used to supply accurate sampling position sites. The map of the sampling locations is presented in Figure 2. These treatment wastewater works are located across the Durban and UmLazi catchment areas under the eThekwini Metropolitan Municipality, KwaZulu-Natal, South Africa. The Southern Wastewater Treatment Works (SWWTW) is located at Wentworth Valley, Bluff and receives the majority of its raw sewage effluent through three large (1500 mm diameter) trunk sewers, i.e., Main Southern (“Jacobs”), Wentworth Valley, and UmLaas Trunk Sewers. Other smaller diameter pipelines coming to the plant include those from Mondi, SAPREF, and Illovo. The total average daily flow to this plant is ±130 mL/day. The UmLaas Trunk Sewer, serving Chatsworth and UmLazi, is predominantly domestic in origin with a discharge flow of ±35 mL/day. This plant discharges all its treated flows directly to the sea through a 4.2 km long, 1500 mm diameter sea outfall [36]. The Isipingo Wastewater Treatment Works (WWTWs) is located in the lower catchment Malukazi Malagazi UmLazi. The facility was built in the late 1960s, collects its raw sewage from the domestic communities within its catchment, and discharges an average of 10.98 mL/day of treated effluent into the Isipingo River [37]. The Northern Wastewater Treatment Plant (NWWTP) is located at 199 Johanna Rd, Peter Road, east Durban, with about 35 industries. The plant receives a capacity of about 60,000 L/day with a monthly capacity of 18.27 mL/month [38] and discharges its treated effluent into the uMgeni River. The New Germany Wastewater Treatment Works (NGWWT) is located at Unit 2 Devon Centre, Durban, 15 Devon Rd, Germany.

### 2.3. Sample Collection

#### Influent, Effluent, and Sewage Sludge

This research explored four WWTPs in the Durban metropolis, KwaZulu-Natal, South Africa. The WWTPs where samples were collected were Isipingo WWTP, Southern WWTW, New Germany WWTP, and Northern WWTP. Influent, effluent, and sludge samples were collected from each of these WWTPs. Raw wastewater (influent), sludge, and final effluent samples were collected at three different points at varying time periods from each WWTPs to ensure a fair mixed representation of the samples. A composite sample was obtained by mixing the samples from the three points. The sampling campaign was done from 15 to 21 September 2021 between 8:15 to 10:35 a.m.; 250 mL influent and effluent samples were collected from each point to make approximately 750 mL after mixing per sampling point. Water samples for TCS and TCC were collected using an amber glass bottle to avoid leaching of phthalate esters that could introduce an extraneous material and interfere with the analytes of interest. Water physicochemical parameters such as pH, electrical conductivity, total dissolved solids (TDS), and salt were measured on-site using a portable multi-parameter meter (HANNA Instrument Inc., HI 9828 pH/ORP/EC/DO, Woonsocket, RI, USA, made in Romania). Samples were kept cool in a cooler box and transported safely to the laboratory. Upon transport to the laboratory, the influent and effluent samples were filtered through joint suction filtration glass Buchner funnel conical flask filters, and stored in a refrigerator at 4 °C prior to further analyses at the Department of Chemical Engineering, Mangosuthu University of Technology, UmLazi. Sludge samples are dewatered using centrifugation, as described by Lozano et al. [39], at 450 r/min for 45 min (HERmLE Labortechnik GmbH, Siemensstr. 25 D-78564 Wehingen, Germany). The supernatant was air dried in the fume hood.

### 2.4. Sample Extraction and Analysis

Filtered wastewater samples were subjected to liquid–liquid extraction as reported by Lehutso et al. [17], but with slight modifications. In this case, a 250 mL filtered wastewater sample was extracted three times (in three cycles) using 20 mL dichloromethane (DCM). The amounts of solvent used in this study were reduced to minimize the level of pollution associated with a chemical solvent being recycled back into the environment. In addition, the pH was left unadjusted as the environmental pH of the wastewater samples collected in this study ranged from 6.06 to 6.65 (Table 1), although Lehutso et al. [17] reported the extraction pH of 6–9 for TCS and TCC. This pH was maintained due to the hydrophobicity nature of TCS and TCC because, at elevated pH, there could be a possible transformation of TCS into its by-products, which could give false information about the real levels of TCS in the samples. Extracts were combined filtered and evaporated to dryness in a fume hood. This was done to maintain the integrity of the analytes and prevent them from being degraded into their by-products while being subjected to elevated temperatures. The dried samples were kept in the fridge (4 °C) before further analysis. Dewatered, dried sludge samples were extracted using the ultrasonication (Ultrasonic Cleaner Model: PS-30, China) method with 10 mL DCM and this was done in three cycles (three times) at a temperature of 30 °C for 30 min each. The sample extract was concentrated following the procedure used for the wastewater samples. As a result of the heterogeneous nature of wastewater samples and to reduce the interferences due to the matrix effect, sample extract was subjected to the clean-up process as described by Lehutso et al. [17], while DCM was used as an eluting solvent for TCS and TCC. Quantification for TCS and TCC was performed using a spectrometer (Shimadzu QP-2010 Ultra, Japan), with a DB-5MS capillary column of the length of 30 m (0.25 μm internal diameter and 0.25 μm film thickness). The GC/MS conditions were: The injector and the transfer line temperature were set to 280 and 300 °C, respectively. The temperature program was initiated at 75 °C for 1 min, increased to 230 °C at 10 °C, then to 280 °C at 20 °C/min, and held for 15 min. The SIM mode used the ions at the targeted ions of m/z 127, 161, 187, 218, 288, 290, and 314. These GC-MS parameters were able to separate the targeted TCS and TCC effectively using the external calibration method. The analytes were identified by comparing their retention times with those of the TCS and TCC standards with their respective base peak ions. A full scan mode was run for qualitative determination of other contaminants of concern that might be present in the wastewater extract in this study. The most prominent compounds other than TCS and TCC were identified by comparing their m/z value with the instrument library, as presented in Table 4.

Nutrients such as ammonium, nitrate, nitrite, phosphate, and total chloride were determined in wastewater samples using a nutrient analyzer (Thermos Scientific Gallery Discrete Analyzer).

### 2.5. Quality Control Measures

The use of solid and liquid soap and antibacterial-coated papers were carefully avoided as much as possible for cleaning any of the glassware during sample preparation. All the sample preparations were done within 72 h. Laboratory blanks were prepared alongside the real sample in the case of external influence, which could introduce the analytes of interest into the samples, thereby, leading to overestimation of a result. The purities of 99.7%, 99.5%, and 95% HPLC grade of DCM, acetone, and n-hexane, respectively, were used in this study. To evaluate the effectiveness of the method for the sample preparation procedure, recovery studies were undertaken. The recovery methods were done by spiking a 5 µg/mL containing TCS and TCC solution in 250 mL distilled water. The solution mixtures were equilibrated overnight and treated as performed for the real wastewater samples. The percentage recoveries were found to be 92%, and 85%. The limit of detection and quantifications were evaluated using three (3) and ten (10) times the standard deviation of the blank with a slope of the regression line respectively. The obtained values were 0.0442 and 0.142 µg/L for TCS and 0.134 and 0.430 µg/L for TCC, respectively. During each analysis in this study, freshly calibration standards were prepared for each set analysis and the regression equation and relative response values were y = 415,949x – 51,010 and R^2^ = 0.9996, respectively.

### 2.6. Ecological Impact Assessment

In this study, an ecological risk assessment was determined. The risk quotient (RQ) and hazard index (RI) were adopted [40] to assess the potential risks TCS and TCC could pose to the aquatic environment when the treated effluent was discharged into the surface water. The measured concentrations of TCS and TCC in the sample obtained in influent and effluent samples collected across four wastewater treatment plants within the Durban metropolis were used for the RQ evaluation in this study. The predicted no effect concentration (PNEC) adopted from previous studies conducted in South African rivers was adopted in this study [41,42]. The RQ and RI values were obtained using Equations (1) and (2), respectively:

(1) RQ=ECPNEC×DF

(2) HI=∑RQ 

where EC is the environmental concentrations of TCS and TCC in the influent and effluent samples, PNEC is the predicted no effect concentration, and DF is the dilution factor when the effluent is being discharged into the surface water systems, which in this case was taken to be 3 [41].

## 3. Results and Discussion

### 3.1. Wastewater Sample Physicochemical Parameters

The wastewater physicochemical parameters and nutrient composition of the influent and effluent samples collected across the Durban metropolis are presented in Table 1.

**Table 1:** Wastewater physicochemical properties and nutrients.

Parameter	Sites/Reference									
	ISW 1	ISW 2	SWW 1	SWW 2	SWW 3	NGW 1	NGW 2	NWW 1	NWW 2	WHO Limit
Ammonia (mg/L)	30.15	3.93	37.70	29.10	41.66	32.43	30.74	36.86	40.80	0.25–32.5
Chloride mg/L)	59.14	57.86	125.14	64.97	270.41	82.20	80.18	68.06	79.31	200–250
Phosphate (mg/L)	3.43	2.49	2.56	3.29	7.22	0.67	0.88	4.82	8.20	0.05–0.10
Conductivity (µs/cm)	702	565	1220	813	1164	1059	802	901	982	≤400
TDS (mg/L)	344	277	598	399	571	519	393	442	482	≤300–600
pH	6.47	6.52	6.23	6.40	6.05	6.14	6.44	6.44	6.65	6.6–8.5

ISW 1, Isipingo wastewater influent; ISW 2, Isipingo wastewater effluent; SWW 1, Southern wastewater domestic influent; SWW 2, Southern wastewater industrial influent; SWW3, Southern wastewater industrial effluent; NGW 1, New Germany wastewater influent; NGW 2, New Germany wastewater effluent; NWW 1, Northern wastewater influent; NWW 2, Northern wastewater effluent.

Ammonia, chloride, and phosphate ions are among the nutrients evaluated in this study. Notably, the amount of ammonia recorded in this study ranged between 29.10 and 36.86 mg/L and between 3.93 and 40.80 mg/L for influent and effluent samples, respectively. The primary sources of ammonia in wastewater are raw domestic, industrial sewage, or agricultural runoff [43]. Elevated ammonia concentrations in wastewater can occur when the decomposition of organic material forms during the decomposition of proteins, manure and urine wastes, and other nitrogen-containing compounds under anaerobic conditions. At low and neutral pH, ammonia exists predominantly as ammonium ions. Elevated concentrations of ammonia can result if excessive nutrients are released into a water body, therefore, causing eutrophication (algae bloom) and depleting the amount of dissolved oxygen [43,44]. A small concentration of ammonia may need up to 4.5 mg of oxygen to oxidize 1 mg of ammonia completely to nitrogen, thereby, causing significant deterioration to the flora and fauna in a body of receiving water [44]. It might also give rise to nitrite formation in distribution systems that may result in taste and odour problems in water. Ammonia is more toxic under alkaline than neutral conditions but has very low toxicity under acidic conditions [45]. The environmental limits for ammonia in surface water in the USA have been set to range from 0.25 to 32.5 mg/L [46]. In addition, a drinking water standard of 0.5 mg/L was recommended by The National Academy of Science and has been adopted by many European nations. The values of ammonia recorded for most of the effluent in this study are far above the recommended value, which could pose a danger to aquatic ecosystems as most of these WWTPs discharged their final effluents into nearby surface rivers. Phosphate in sewage effluent arises from digested food and domestic phosphate-based detergent. The effect of phosphate in an aquatic environment is similar to what ammonia could pose, as both encourage the growth of weeds over the growth of other wildlife. The concentrations of phosphate recorded in this study are significantly higher than 1 mg/L, except for the New Germany effluent sample (NGW 2) of 0.67 mg/L as stipulated by the South African standard for phosphate for sewage effluents [47] that will be discharged into receiving surface water. Chloride is one of the most frequently used disinfectants for treating municipal wastewater due to its ability to destroy pathogenic organisms capable of oxidizing cellular materials. Its usage has been due to minimal health implications and safety limitations with a long history of being an effective disinfectant, although residual chlorine at low concentrations can be toxic to aquatic life [46]. The levels of chlorine obtained in the influent across the WWTPs in this study ranged from 59.14 to 125.14 mg/L, while effluent chlorine levels ranged from 57.86 to 270.41 mg/L. It should be noted that the levels found in the influent samples do not show any significant change from what was present in the discharge in almost all of the plants investigated in this study. The data in this study suggest that the levels present in the effluent samples may probably reflect the addition of chlorine being used as one of the treatment approaches to mitigate the microbial or pathogenic loads in raw wastewater before being discharged into surface water. The WHO has set the tolerable level of 200 mg/L for chlorine in drinking water and South Africa has chlorine limits of 250 mg/L for drinking water. The levels recorded across the sites in this study were lower than the set limits, except for SWW3 which recorded a level of 270.41 mg/L. This WWTP receives its raw wastewater from both domestic and industrial sources where the chlorine level could reasonably be higher due to the complexity of several industrial chlorinated containing materials being treated. This particular plant discharges its effluent into the ocean. Consequently, an elevated chlorine level arising from waste effluents can upscale the salinity of the water, thereby, resulting in adverse ecological effects on the biota in the aquatic environment [48]. Electrical conductivity (EC) and levels of total dissolved solids (TDS) recorded in this study could suggest a direct relationship of other nutrients such as ammonia, phosphate, and chloride, which could probably result from various organic materials and ions. EC is the measure of water capacity to conduct an electric charge, which depends on dissolved ion concentrations, and ionic strength at a particular temperature at the measurement was carried out [49,50,51], whereas, TDS is usually measured as the concentration of dissolved ions. Domestic, industrial waste, and agricultural runoff may be potential sources of material for TDS and EC. The EC and TDS values for effluent samples in this study ranged from 565 to 1164 µs/cm and from 277 to 571 mg/L, respectively. The ratio of TDS to EC (TDS/EC) for sewage effluent in this study was between 0.490 and 0.491, which was favorably below the accepted limits of 0.55 by the WHO standards for natural and freshwater systems [49,52]. However, EC in the effluent samples exceeded the WHO recommended value of 400 µs/cm.

### 3.2. Levels of TCS and TCC in Sewage Influent and Effluent

**Figure 3:**
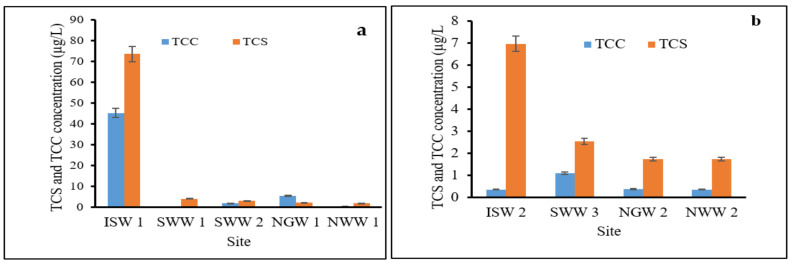
Concentrations of TCS and TCC in wastewater influent and effluent samples. (**a**) concentrations of TCS and TCC in the influent samples; (**b**) concentrations of TCS and TCC in the effluent samples.

Due to their frequent usage, the environmental implication to aquatic life, and the health-associated effects of TCS and TCC on humans, it is crucial to evaluate the levels of TCS and TCC from raw sewage and effluent that finally end up in surface water. Four selected WWTPs across the Durban metropolis were sampled in this study. These WWTPs receive their raw sewage from domestic sources and a few receive from both domestic and industrial sources. Raw wastewater and effluent were both evaluated for contaminants of concern in this study; the results are presented in Figure 3a,b respectively.

On the one hand, the concentrations of TCS in the influent and effluent ranged from 1.91 to 73.50 µg/L and from 1.732 to 6.980 µg/L, respectively. On the other hand, concentrations of TCC in the influent and effluent ranged from 0.589 to 45.30 µg/L and from 0.344 to 1.103 µg/L, respectively. The results indicated that substantial amounts of TCS and TCC are present in the samples. Based on the distributions of TCS and TCC across the sampling point, it was noted that the contaminants of concern were present at levels above the detection and quantification limits (LOD and LOQ) of 0.177 µg/L and 0.536 µg/L, except for the TCC in the SWW1 that received its waste from both domestic and industrial sources. At this point, the concentration of TCC in the domestic influent was 0.321 µg/L, which was below the LOQ. TCC at the Isipingo, New Germany, and Northern wastewater effluent samples had concentrations of 0.344 µg/L, 0.372 µg/L, and 0.348 µg/L, respectively, which were well below the LOQ. Among the waste treatment plants, the Isipingo plant was noticed to have the highest levels of both TCS and TCC in its influent sample followed by New Germany, which also recorded a reasonable level of TCC in its sewage influent sample (5.554 µg/L). The possible scenario for this observation could be that the treatment plants received a higher volume of waste from personal care and consumer products (PCPs) that contain higher percentages of these contaminants than other WWTPs. In addition, the consumption patterns of PCPs with TCS and TCC formulation among the residents within their respective locations or catchments could be responsible for the elevated concentrations observed at these locations. Other important factors for the increase in the concentration at these plants could be variations in the consumption patterns, types, volume and composition of waste streams being received, treatment technologies, and effectiveness of the WWTPs adopted [17,53]. From the values recorded in this study, it could be deduced that there was a sharp decline in the levels of TCS and TCC recorded in the influent to effluent samples across the sites. This could be because of the substantial amounts of antimicrobial load in the influent samples, which are yet to be subjected to any forms of treatment for their removal. It should also be noted that the treatment procedures adopted in these plants are capable of removing substantial amounts of antimicrobial agents (TCS and TCC) from the waste sludge, and therefore, the remaining effluent has low levels of these contaminants. The trend recorded in this study was similar to that reported by Kumar et al. [54], who reported a significant decline in the concentrations of TCS and TCC from 5213 to 86,161 ng/L, from 180 to 5370, from 3505 to 36,221 ng/L, and from 281 to 3034 ng/L, for influent and effluent, respectively, in the samples collected across four wastewater treatment plants in Georgia, USA. A similar observation was also reported from a study conducted in selected wastewater treatment plants in Gauteng Province, South Africa by Lehutso et al. [17]. TCS concentrations of 2.01–17.6 µg/L were reported in the influent samples, whereas effluent samples had a concentration range from 0.990 to 13.0 µg/L. A similar pattern was seen for TCC with a concentration between 0.0860 and 2.84 µg/L and below LOD–1.89 µg/L in influent and effluent samples, respectively. Another study by Heidler et al. [55], from the east coast of the United States, reported a sharp decline from 6.1 to 0.17 µg/L of TCC in the influent and effluent samples, respectively. Higher concentrations of TCS (2.237–73.462 µg/L) were found in influent and effluent samples across the site than TCC (0.320–45.261 µg/L) in this study. The results of this study are consistent with what has been reported in the literature [17,54]. Tran et al. [25] also reported the occurrence and fate of emerging contaminants in municipal wastewater treatment plants from different geographical regions. The results indicated that from 1.3 to 86,200 ng/L and from 3.1 to 5370 ng/L, for TCS and TCC, respectively, were found in the influent samples investigated. The possible reasons could be attributed to the higher water solubility of TCS than TCC or TCS has a higher composition as an antimicrobial agent in PCPs, which are more frequently used within the catchment where these WWTPs receive their sewage influent [25,53].

### 3.3. Levels of TCS and TCC in Sludge Samples 

**Figure 4:**
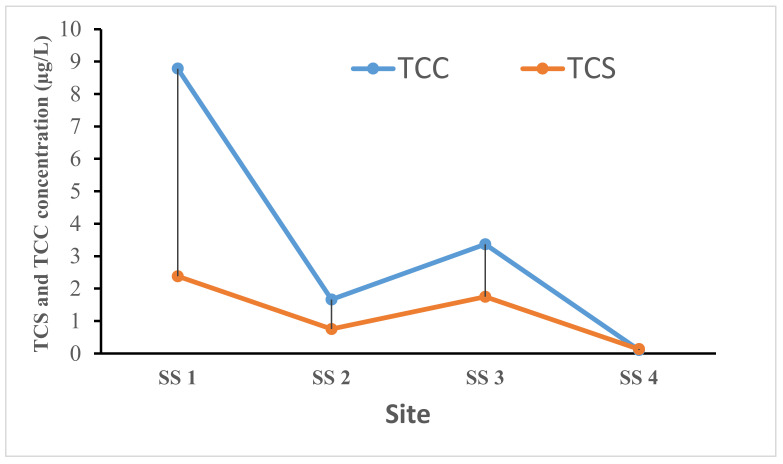
Distribution of TCS and TCC in wastewater sludge. (SS 1, sludge sample at Isipingo WWTP; SS 2, sludge sample at Southern WWTP; SS 3, sludge sample at New Germany WWTP; SS 4, sludge sample at Northern WWTP).

The levels of TCS and TCC found in the sludge samples collected across the selected WWTPs in this study are presented in Figure 4. The concentrations of TCS and TCC found in the sludge samples ranged from 0.136 to 2.382 µg/kg and 0.107 to 8.782 µg/kg. The concentration of TCC was significantly higher than the level of TCS across the sampled WWTPs except for the Northern wastewater treatment plant (SS 4). The observed trend here could be due to its relatively low water solubility and high logarithmic octanol/water partitioning coefficients (log K_ow_). These properties typically enable TCC to have substantially higher antimicrobial concentrations in wastewater sludge as compared with TCS. TCS has high water solubility (10.0 mg/L) with a pKa of 7.9 and is prone to microbial degradation, preferentially being converted into its metabolites through biotic and abiotic processes and mechanisms within organisms and WWTPs as well as in the environment [20,56,57]. Whereas only a fraction of TCC in wastewater is removed through biologically mediated transformation and as a result of its molecular nature. TCC can remain unionized in a broad range of pH values (pKa = 12.7) and displays a more limited activity as compared with TCS and [21,22], although TCC can undergo both chemical and biological transformation processes due to its trichlorinated structure, thereby, forming chlorinated anilines which are hematotoxic and carcinogenic as compared with the parent TCC [58].

The concentrations of TCS and TCC in sludge samples were both generally higher than those found in filtered influent samples. This could primarily be due to their sorption capacity and relatively high hydrophobic character [53,58,59,60]. More importantly, TCS and TCC have a higher tendency to partition onto waste sludge and are removed during treatment processes, thereby, leaving the effluent with a low percentage of TCS and TCC before being discharged into nearby surface waters. The results of our study were compared with the global reported concentrations of TCS and TCC in wastewater influent, effluent, and sludge samples, as presented in Table 2. Zheng et al. [61] evaluated the concentrations of TCC from four WWTPs in Zhengzhou, China; the results indicated that the concentration of TCC in the influent sample was 0.731–0.812 µg/L, while the sludge sample had a concentration of 1430.1–1663.8 µg/kg. This was far higher than that found in the influent and sludge samples in our study, although similar trends were reported for influent and sludge TCC composition. Additionally, Zhu et al. [62] investigated the spatial distribution of TCC in sewage sludge in China where a mean concentration of 2350 µg/kg was reported which was higher than that found in our study. The concentrations of TCS and TCC found in our study were relatively lower as compared with that reported in samples collected in selected wastewater treatment plants in Gauteng Province, South Africa [17]. The authors noted that the concentrations of TCS ranged between 3.70 and 15.0 µg/kg for raw sludge and the TCC concentrations were in the range of 4.12–11.8 µg/kg. The reported concentrations were higher than those found in our study. Furthermore, Guerra et al. [8] investigated the levels of TCS in the wastewater samples collected in Ontario, Canada. Concentration ranges of 0.289–33.500 µg/L and 0.030–1.390 µg/L for influent and effluent, respectively, were reported. The levels found were lower as compared with those reported in this present study, although similar trends were observed in both influent and effluent samples. Tran et al. [63] conducted a similar study in Singapore where lower concentrations of TCS and TCC as compared with this present study were reported. The concentrations of TCS and TCC ranged from 0.341 to 0.744 µg/L, from 0.0285 to 0.046 µg/L, from 0.424 to 0.934 µg/L, and from 0.143 to 0.215 µg/L for influent and effluent samples, respectively.

### 3.4. Ecological Risk Assessment of TCS and TCC

The ecological risks of TCS and TCC in influent and effluent samples collected from four different wastewater treatment plants across the Durban metropolis using RQ were evaluated. It is important to know the risks the discharge of the final effluent could pose when the pollutants are not efficiently removed during the treatment processes from wastewater plants. The values of RQ are presented in Table 3.

According to the risk ranking criteria, RQ < 0.01, 0.01 < RQ < 0.1, 0.1 < RQ < 1, and RQ > 1 indicated minimal risk, low risk, medium risk, and high risk, respectively [40,67,68]. The RQ values for TCS, TCC, and RI from the Isipingo wastewater influent are 1.75 × 102, 6.036 × 101, and 1.17 x 101, respectively. It should be noted that these values are greater than one, which indicates that raw sewage from this wastewater plant finds its way directly into the receiving surface water (Isipingo River). This could lead to a very serious ecological problem or appreciable risk could exist in the environment, although RQ values for influent samples across other wastewater plants were found to be below one, which indicated that ecological risk to the aquatic environment was considered to be unlikely. The RQ values in influent and effluent samples from the Isipingo wastewater plant decreased significantly from 17.5 to 1.66 and from 6.04 to 0.046 for TCS and TCC, respectively. Similar trends were observed across the other wastewater plants where the RQ values were generally less than one. The RQ values for TCS were also found to be higher than that of TCC.

A similar observation was recorded by Musee [41] where the RQ values in the influent and effluent samples were reported to be from 9.4–31 to 1.4–12.9 and from 0.6–0.7 to 0.1–0.3 for TCS and TCC, respectively. Zeng et al. [61] also noted that the RQ value of TCC was significantly reduced from 6.17 in the influent sample to 0.31 in the effluent sample. This indicated that the wastewater treatment processes effectively reduced the risk of TCS and TCC in the effluent and minimal to low risk was possible. The trend could also be that TCS is more frequently used in household products than TCC or TCS is more toxic than TCC, which poses more of a threat to organisms in contact with aquatic environments than TCC.

### 3.5. Other Important Identified Compounds Found in the Wastewater Samples in This Study

In this study, extracted filtered wastewater samples were qualitatively analyzed for any possible contaminants of concern (CECs) using GC-MS. The notable compounds qualitatively identified are presented in Table 4. The compounds identified are caffeine (C_8_H_10_N_4_O_2_), tert butylhydroquinone (C_10_H_14_O_2_), chloroxylenol (C_8_H_9_OCl), phenol, 4-(1,1,3,3-tetramethyl butyl) (C_14_H_22_O), and dimethyl-bisphenol A (C_17_H_20_O_2_). The details of these compounds in terms of their properties, sources, and application, as well as environmental and health impacts are well documented in Table 4. The identified compound in wastewater influent and effluent samples are crucial due to their environmental and health implications to aquatic life and the health of humans who might have contact with the effluent from these WWTPs which is discharged into the closest rivers and used for various purposes such as domestic, agricultural, and horticultural activities along the course of the rivers.

## 4. Conclusions

The findings of this study are important due to the crucial nature of the health-related issues of TCS and TCC to aquatic environments and human health. TCS and TCC enter the aquatic environment through wastewater and effluent discharge pathways. In this study, influent, effluent, and sludge samples collected in selected wastewater treatment plants across the Durban metropolis were quantitatively investigated to determine the concentration levels of TCS and TCC. In addition, samples were qualitatively analyzed to identify any possible contaminants of concern. The results of this study revealed that TCS and TCC were present in the samples analyzed. It was observed that the concentrations of TCS ranged from 1.906 to 73.462 µg/L, from 1.732 to 6.980 µg/L, and from 0.138 to 2.455 µg/kg in influent, effluent, and sludge samples, respectively. The concentrations of TCC were found to be between 0.320 and 45.261 µg/L, <LOQ–1.103 µg/L, and from 0.107 to 8.827 µg/kg in the influent, effluent, and sludge samples, respectively. The concentrations of TCS were found to be higher in the aqueous samples as compared with those recorded for TCC in the same medium. However, the concentrations of TCC in the sludge samples were significantly higher than the levels recorded for TCS. The trend observed could be due to the higher aqueous solubility of TCS and more hydrophobicity character of TCC. Another possibility could be that TCS is more frequently applied as an antimicrobial agent in personal care and consumer products. Generally, the mean concentrations of TCS and TCC in influent and sludge samples were both higher than those found in the effluent samples across the treatment plants investigated. In addition, the risk quotient was evaluated in the influent and effluent samples. The RQ values in the influent samples were generally lower than those found in the effluent samples. The RQ value reported for TCS was found to be significantly higher than TCC; therefore, TCS could pose a greater threat to organisms in contact with aquatic environments than TCC. It could be assumed that the treatment processes adopted by the treatment plants were able to remove substantial amounts of TCS and TCC from the raw sewage waste before being discharged to nearby surface rivers, thereby, reducing the ecological risk that could be associated with TCS and TCC in the environment. The qualitative investigation revealed that some endocrine xenobiotic compounds such as caffeine, tert butylhydroquinone, chloroxylenol, phenol, 4-(1,1,3,3-tetramethyl butyl), and dimethyl-bisphenol A were also present. To safeguard aquatic environments and the health of people depending on the receiving rivers across the catchment areas, it is important for frequent monitoring of the levels of these contaminants of concern and to identify the most effective treatment methods to remove these xenobiotics and endocrine-disrupting compounds in raw sewage wastewater before being discharged. There is also a need for proper and quantitative investigations of the levels of caffeine, tert butylhydroquinone, chloroxylenol, phenol, 4-(1,1,3,3-tetramethyl butyl), and dimethyl-bisphenol A in raw sewage and effluent samples, as these compound are more toxic and pose serious health-related issues for humans and aquatic environments.

It is believed that the data generated and the outcomes of this study would provide the much-needed information that could be useful for the relevant agencies on the levels and fate of the targeted pollutants. Due to the crucial nature of this study’s subject matter, the outcomes of this study should propel further studies for the best possible remediation approach for the targeted and other potential emerging contaminants in wastewater for safeguarding the receiver surface water along with the point of their discharge.

## Figures and Tables

**Table 2:** Concentration of TCS and TCC in wastewater influent, effluent, and sludge samples reported across the globe.

Literature	TCS			TCC		
	Influent (µg/L)	Effluent (µg/L)	Sludge (µg/kg)	Influent (µg/L)	Effluent (µg/L)	Sludge (µg/kg)
This study (2022), Durban, S.A	1.906–73.462	1.732–6.980	0.138–2.455	0.320–45.261	<LOQ–1.103	0.107–8.827
[64], Luxembourg	0.02 -86.161	0.023–5.370	580–15600	-	-	-
[8], Canada	0.289–33.500	0.030–1.390	-	-	-	-
[62], China	-	-	-	-	-	2350.0
[17], S.A	2.10–17.60	0.990–13.00	3.70–15.00	0.0860–2.84	<LOQ–1.89	3.65–11.8
[65], North Texas, USA	26.8	0.25	-	-	-	-
[61], Zhengzhou, China	-	-	-	0.731–0.812	-	1430.1–1663.8
[55], USA	-	-	-	6.10	0.170	-
[66], Paris, France	-	-	-	0.097–0.140	BDL	-
[63], Singapore	0.341–0.744	0.0285–0.046	-	0.424–0.934	0.143–0.215	-

**Table 3:** Risk quotient (RQ) and risk index (RI) values for TCS and TCC in raw influent and final effluent samples.

Sample	Influent RQ					Effluent RQ			
	ISW 1	SWW 1	SWW 2	NGW 1	NWW 1	ISW 2	SWW 3	NGW 2	NWW 2
TCC	6.036 × 10^1^	4.30 × 10^−2^	2.61 × 10^−1^	7.41 × 10^−1^	7.90 × 10^−2^	4.60 × 10^−2^	1.47 × 10^−1^	5.0 × 10^−2^	4.60 × 10^−2^
TCS	1.75 × 10^2^	9.82 × 10^−1^	7.23 × 10^−1^	5.33 × 10^−1^	4.54 × 10^−1^	1.66 × 10^1^	6.05 × 10^−1^	4.15 × 10^−1^	4.12 × 10^−1^
RI	1.17 × 10^1^	5.12 × 10^−1^	4.92 × 10^−1^	6.37 × 10^−1^	2.66 × 10^−1^	8.54 × 10^−1^	3.76 × 10^−1^	2.32 × 10^−1^	2.23 × 10^−1^

**Table 4:** Other qualitatively identified compounds in wastewater samples.

Identified Compounds	Properties	Sources and Application	Environmental and Health Impacts
Caffeine (C_8_H_10_N_4_O_2_) 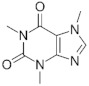	Caffeine is a potent stimulant with a direct effect on the central nervous system. It has a high water solubility of 20.17 g/L (25 °C)	Caffeine is commonly found across the world in some plant parts such as seeds, fruits, nuts, or leaves [69]. Caffeinated drinks and foods also contain substantial amounts of caffeine and are used widely as an ingredient in some over-the-counter medications such as analgesics, stimulants, illegal drugs, and cold medicines [70]. It enters surface water primarily through wastewater effluent through anthropogenic sources [71].	When present within the environmentally relevant concentrations, caffeine can pose serious effects on aquatic life. Such effects include lethality, decreasing general stress, inducing oxidative stress and lipid peroxidation, affecting energy reserves and metabolic activity, neurotoxic effects, and affecting reproduction and development [72].
Tert butylhydroquinone (C_10_H_14_O_2_) 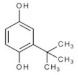	Tert butylhydroquinone is a good antioxidant that is extensively used as a preservative. It is soluble in water less than 1 g/L (18 °C).	Tert butylhydroquinone finds wider application as a fixative agent in perfumery. It is an important agent commonly applied as an antioxidant in biodiesel and used as a stabilizer to inhibit autopolymerisation of organic peroxides [73]. The industrial effluent discharge could serve as a point source to TBHQ into the surface water or leach into the groundwater through sludge, which is used as fertilizer.	Tert butylhydroquinone may be carcinogenic when expose to prolonged very high doses. (Gharavi and Kadi [74], especially for stomach tumors [75]). Reports had also shown that exposure to tert butylhydroquinone could result in visual disturbances. [76].
Chloroxylenol (C_8_H_9_OCl) 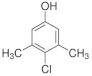	Chloroxylenol is an antimicrobial agent commonly used in many cosmetic products. Freely soluble in an organic solvent but fairly soluble in water (0.03 wt%).	The dermal and gastrointestinal tract is the common route of exposure to chloroxylenol in humans. It is frequently used as a disinfectant and for sanitation in hospitals and households. Other important applications are antibacterial soaps, wound-cleansing applications, and household antiseptics [77]. A recent investigation has shown products containing chloroxylenol are effective against the SARS-CoV-2 virus [78]. The use of chloroxylenol in these products can end up in surface water through sewage generated from domestic, industrial, and hospital waste.	Chloroxylenol has been reported to be moderately toxic to freshwater invertebrates and highly toxic to fish [79]. It can generate active species that can cause cancer [80].
Phenol, 4-(1,1,3,3-tetramethylbutyl) (C_14_H_22_O) 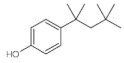	Phenol is commonly produced via a catalytic reaction involving phenol and diisobutylene at high temperatures. It has a high water solubility of 19 mg/L at 22 °C.	The main route of phenol, 4-(1,1,3,3-tetramethyl butyl) is produced through the catalytic reaction of phenol with diisobutylene at temperatures from 80 to 100 °C in a closed system. Octylphenol ethoxylates are used as a surfactant in detergents and cleaning agents, and maybe degraded back to its by-products in an aquatic environment.	Its acute toxicity to human health with slight skin irritation could be highly irritating to the eyes. It may lead to skin depigmentation but is not genotoxic. Displacement of 17-β-estradiol from its receptors in a competitive manner is possible and it can also promote cell proliferation in estrogen-dependent cells [81].
Dimethyl-bisphenol A (C_17_H_20_O_2_) 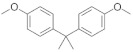	Dimethyl-bisphenol A is an industrial chemical that has been extensively used for the production of certain plastics and resins since the 1950s. It has a moderate water solubility of 120–300 mg/L at 25 °C)	Dimethyl-bisphenol A is essentially found in polycarbonate plastics and epoxy resins. Diet has been a major exposure route to dimethyl-bisphenol A for humans [82]. Other possible sources of exposure include air, dust, and water. A larger percentage of dimethyl-bisphenol A exposure occurs through daily human food and beverages [83]. Its moderate water solubility (120–300 mg/L at 25 °C) enables either its release into the effluent from domestic or industrial manufacturing units using bisphenol A-based products.	It is an endocrine disruptor. It can also imitate body hormones in a way that could be hazardous to health. Its impacts on humans include cardiovascular problems [84], reproductive effects [85], mammary gland and developmental problems, low sperm production, and fetal growth restriction [85,86,87]. Other effects include anxiety and depression, obesity [88,89], hormone-related cancers such as breast cancer or prostate cancer, and allergic contact dermatitis [86,90].

## Data Availability

Data related to this article can be obtained from the department of Chemical Engineering at the Mangosuthu University of Technology (https://www.mut.ac.za/chemicalengineering/, accessed on 20 April 2022) or via authors adeyinka.gbadebo@mut.ac.za; bfemi@mut.ac.za.
